# Prevalence of dehydration and a quality improvement initiative in nursing home residents

**DOI:** 10.1016/j.jarlif.2026.100084

**Published:** 2026-08-22

**Authors:** Irene Hamrick, M. Lisa Bauer

**Affiliations:** University of Cincinnati, College of Medicine, Department of Family and Community Medicine, Division of Geriatrics, Cincinnati, Ohio United States

**Keywords:** dehydration, hydration, geriatrics, older adults, institutionalization, nursing home

## Abstract

**Objectives:**

Dehydration in older adults is common and is 37% among community living older adults. No prevalence in US nursing homes is published.

**Primary:**

To describe the prevalence of dehydration, defined as BUN/creatinine ratio ≥20:1, among residents of a single veterans administration (VA) nursing home over three years.

**Secondary:**

To describe concurrent trends in selected quality metrics during the implementation of a multi component hydration quality improvement program.

**Methods:**

We identified dehydration based on blood urea nitrogen and creatinine (BUN/Creat) ratio as ≥20:1 because of convenience and availability.

We implemented several approaches to improve dehydration in our VA facility: Providing favorite drink at: bedside, table or wherever resident is.

Encourage to drink on entry to room and before leaving,

Flavored water,

Door magnet,

Huddle meetings with staff:

Education of aging changes that put older adults at risk of dehydration

Rationale for need of 2 quarts or more a day

Soliciting from frontline staff for their tips on how to increase fluid intake.

Asked for barriers to implementation,

Discussing with staff and showing study progress over time.

**Results:**

Of 20-48 residents (varied because of pandemic) between 2021-2023:

Dehydration improved from 75% to 36.6% over 3 years.

We found associations with:

Improved behaviors in our residents and fewer hospitalizations.

Fall rates declined every year, from 6.4 in 2021 to 3.9 in 2023.

UTI decreased, and our catheter associated UTIs moved our facility to the 1^st^ quintile, receiving a #1 in the nation ranking among all VA nursing homes.

Challenges: Resident turnover, co-morbidities, poor insight, and staff turnover requires us to keep education ongoing.

**Conclusion:**

Multiple approaches and ongoing education over time cut our dehydration rate in half over 3 years. We noted fewer UTIs, falls, behaviors and hospitalizations. This material is the result of work supported with resources and the use of facilities at the Cincinnati VA Medical Center.

## Highlights

• Dehydration was highly prevalent in our nursing home, at 75%, double that in the community.
• Normal Aging changes contribute to dehydration.
• Several interventions cut the prevalence in half, to 36% over 3 years.
• Staff education and reminders are key to improving dehydration in facilities.
• Multiple resident oriented outcomes were associated with improvement in hydration.

## Graphical abstract

**Figure:**
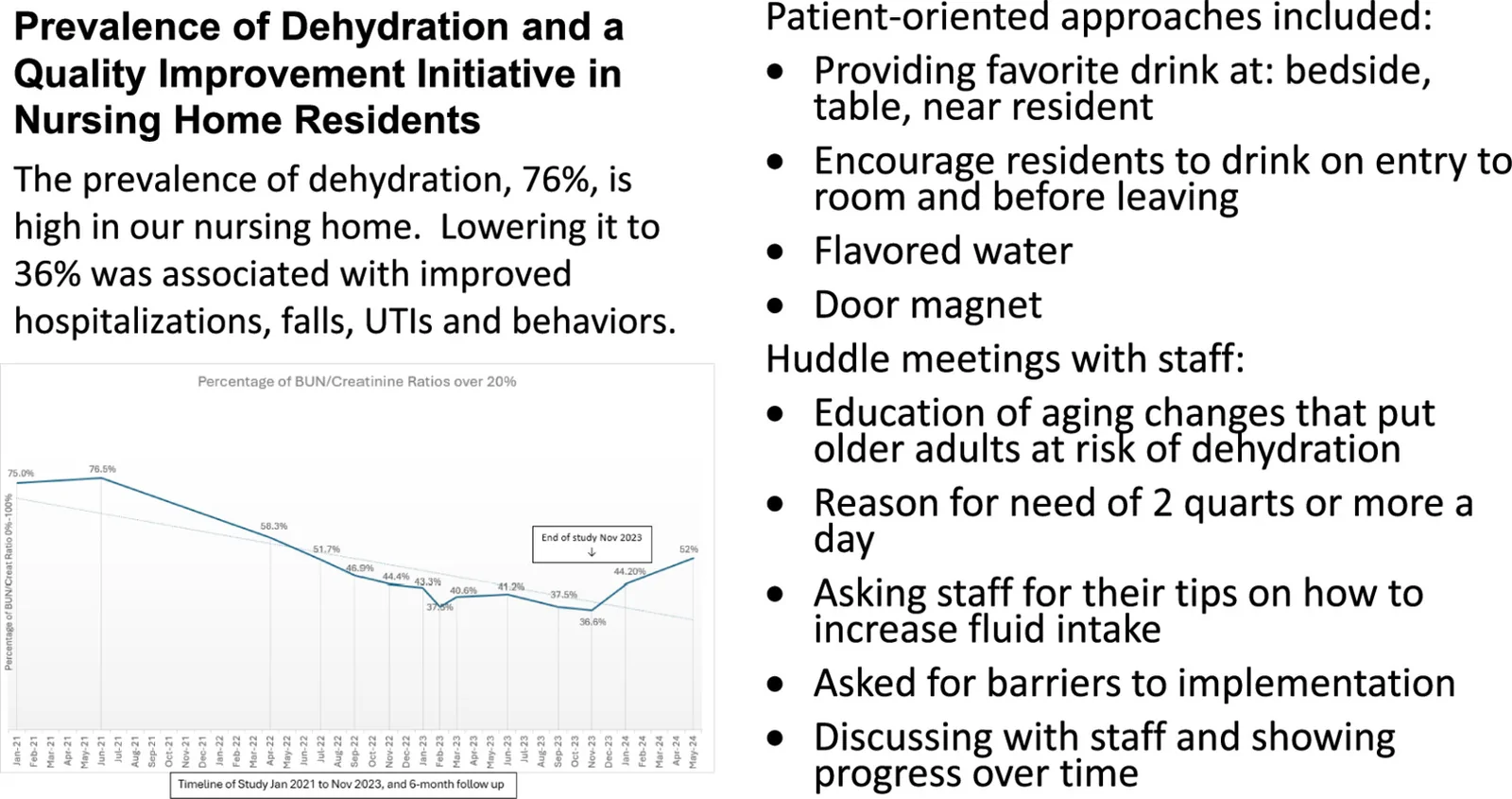

**Abbreviations**
ADH: Antidiuretic hormone
UTI: Urinary tract infection
VA: Veterans administration
QM: Quality measures
SAIL: In strategic analytics for improvement and learning
QAPI: Quality assurance and process improvement
BUN: blood urea nitrogen
SS: Short stay
LS: Long stay
CAUTI: Catheter associated UTIs
CRF: Chronic renal failure
NH: Nursing home
MRSA: Methicillin resistant staph aureus

**Brief Summary**

We found a prevalence of dehydration of 75% in our nursing home residents and describe our various approaches to improve the prevalence to 36% over 3 years. This was associated with improved quality measures of falls, UTIs, and hospitalizations.

Alt-text: Unlabelled box dummy alt text

## 1 Introduction

Several aging changes put older adults at increased risk of dehydration. For instance, apoptosis of neuronal cells in the thirst center in the brain reduce the feeling of thirst for many older adults [1] Similarly, advancing age brings apoptosis, leading to decreased antidiuretic hormone (ADH) production. Less ADH leads to decreased urine concentrating ability and more fluid loss [2] Even if sufficient ADH is available, the senescent kidneys are not as capable of concentrating the urine due to apoptosis of nephrons [3,4] Aging brings the replacement of muscle mass with adipose tissue. Since muscle is the tissue with the highest water content, fluid reserve is further reduced [5,6]

Dehydration is associated with constipation [7] kidney stones [8] delirium [9] urinary tract infection (UTI) [10] falls [11] and fatal coronary heart disease [12] Dehydration can additionally lead to orthostatic hypotension and to impaired brain perfusion [13] Upon standing, the decreased elasticity of aging vasculature leads to decreased venous blood return and lowers diastolic blood pressure [14]

There is a 37.9% prevalence of dehydration in an electronic health record review of 30,634 adults 65 and older, including all settings [11] A study of hospitalized older adults found also that the prevalence of dehydration was 37%, while this rate was twice as high (79%) among those who died during hospitalization [15] dehydration might be a contributor to morbidity and mortality among both hospitalized and non-hospitalized older Americans.

Assessment of dehydration is more challenging in older adults due to the decreased elasticity of the skin which makes skin turgor unreliable. Additionally, mucus membranes may be dry due to decreased production of saliva due to loss of needed tissue from apoptosis [16] The best measurements for dehydration in older adults are serum osmolality, serum blood urea nitrogen/ Creatinine (BUN/Creat) ratio, and serum sodium level [17]

Dehydration prevalence in nursing homes is not reported to our knowledge. This study reports the prevalence of dehydration and reviews the quality improvement project to reduce dehydration in our facility using a multi-component hydration bundle. Our objectives were:

- Primary: To describe the prevalence of dehydration, defined as BUN/creatinine ratio ≥20:1, among residents of a single VA nursing home over three years.

- Secondary: To describe concurrent trends in selected quality metrics during the implementation of a multi component hydration quality improvement program.

## 2 Material and Methods

### 2.1 Study design and data collection

The study is a retrospective chart review over 3 years based on a quality improvement initiative in an urban nursing home.

Our study was approved by the local Institutional Review Board.

### 2.2 Cohort description

The study population consisted of older adults living in a nursing home of predominantly male residents. The number of residents and the average age varied widely between 20 and 48 residents from week to week, due to pandemic related intermittent lockdowns and restrictions and staffing shortages.

The facility has 16 skilled or rehab beds, 1 respite bed, 5 hospice beds and 26 long-term care beds.

The facility had a broad spectrum of common illnesses seen in older adults. Because we used anonymized data, we do not have an account of the number of diagnoses of our residents.

### 2.3 Setting

Midwest 48 bed nursing home facility with skilled, rehab, hospice, long-term care and respite residents.

### 2.4 Intervention

We implemented several approaches to improve dehydration rate in our Veterans Administration (VA) facility. Huddle meetings are 15 min interdisciplinary meetings on each nursing unit, addressing newly admitted residents and residents with new or ongoing issues. Huddles were held 3 times a week with fluctuating attendance due to resident acuity, staffing fluctuations and shortage. Eventually all staff participated in some huddles over the 3 years of the initiative. During the huddles we addressed aging changes that put older adults at risk of dehydration. The medical director addressed aspects of physiologic changes in 1-3 minute presentations during huddles as pertinent to a resident. Over time the various reasons that put older adults at risk of dehydration were addressed, and staff communicated them with each other:

Rationale for need of 2 quarts or more a day, including greater fluid losses due to lower ability to concentrate the urine.
Soliciting from frontline staff for their tips on how to increase fluid intake
Asked for barriers to implementation
Encourage to drink on entry to room and before leaving
Discussing and showing study progress over time
Flavored water
Door magnet showing a glass of water with a straw.

After having the magnets on the door frame for a few weeks, we removed them for fear that the survey team would find them infringing on resident confidentiality.

We used an interdisciplinary Team approach for Process Improvement: Pharmacy, Therapy team, Providers, Quality Nurse, Dietician, Social work and MDS team members each follow quality measures (QM) specific to their levels of expertise and provide detailed causal analysis of factors that trigger each quality metric.

• Weekly real time data review to identify risk factors during interdisciplinary team meetings for pertinent measures, such as falls.
• Monthly review of all 32 QMs in Strategic Analytics for Improvement and Learning (SAIL) during Quality Assurance and Process Improvement (QAPI) meetings.
• Quarterly Reports and analysis of current monthly trends and deep dive analysis into QM metrics each quarter as quality reports are released. Discussions include declining conditions and recent changes:
  Mobility changes
  Medication changes
  Pain
  Increased fall risk
  Recent hospitalizations
  Decline in mental status/ changes in cognition
  Skin assessment changes
  Transition to hospice for end of life
  And all other QM

No change occurred in the initiation, use and care of catheters during the time of the initiative.

### 2.5 Outcome indicators

We collected lab data from the CPRS electronic health records for: blood urea nitrogen (BUN) and creatinine and tracked them over time. The labs were monitored for medication management or acute reasons as needed and varied among residents. At minimum all residents had annual measurements, except hospice residents. We reviewed residents’ labs between 3 and 12 months, see graph due to COVID isolation. The percentage was calculated by elevated BUN/Cr ratio ≥20 divided by the number of residents with the labs measured at the time. We monitored the 15 QMs as reported to CMS and 31 QMs reported to VA in SAIL that are used for VA internal benchmarking.

## 3 Data Analysis

We calculated the percentage of residents found with elevated BUN/Creat ratio ≥20/1 and tracked the results over time.

## 4 Results

Our facility had 20-48 residents of older adults, mostly men, over the 3 years of our quality improvement project from January 2021 to December 2023. Most residents were long-term care, some palliative care and hospice and very few skilled nursing admissions due to pandemic restrictions. The prevalence of dehydration improved from 75% to 36.6%.

Dehydration prevalence at each timepoint was calculated as the number of residents with BUN/Cr ratio ≥20:1 divided by the total number of residents with labs recorded at that measurement time point.

We saw consistent and marked improvement in our QM scores in 11 of 31 internal VA SAIL quality metrics that correlated with our initiative since 2021. There has been a downward trend in NH falls rates since January 2021. The annual fall rates improved from 6.4 in 2021 to 3.9 in 2023. Many veterans are catheter dependent due to spinal cord injury or neurogenic bladder from other causes. For long stay (LS) residents, catheter associated UTIs (CAUTI), we moved from 5^th^ quintile to 1^st^ quintile and have sustained a 1^st^ quintile ranking for the last 4 quarters and were ranked first in the nation, with a rate of 0/1000 catheter days. A reduction in number of indwelling catheter associated UTIs in the NH coincided with our initiative and has remained in the 1^st^ quintile for the 11 quarters since January 2021. For facility-acquired methicillin resistant staph aureus (MRSA) we have moved from the 5^th^ to the 1^st^ quintile over the 11 quarters. We have seen a decline in the number of hospitalizations per 1000 LS resident days and have moved from the 5^th^ quintile to the 3^rd^ since January 2021. And there has been a concurrent decrease in the number of LS UTIs since January 2021, from 15 in FY 2022 to 7 in FY 2023. For Short Stay/Rehab residents SAIL measures – Improvement in Function, rehospitalization after NH admission, and discharged to the community all were associated with continued and consistent improvement in scores over the 11 quarters. See Table 1.

**Table 1:** Improvement of quality metrics over the time of our study.

Quality Metric	2021	2023
Fall rates	6.4	3.9
Long-stay URIs	15	7
Catheter-associated UTI rates	5th quintile	1st quintile
Facility-acquired MRSA infections	5th quintile	1st quintile
Hospitalizations per 1000 long-stay resident-days	5th quintile	3rd quintile

## 5 Discussion

To our knowledge this is the first report of dehydration prevalence in a nursing home. Our biggest challenge was staff turnover. We only briefly had a nurse champion. Showing improvement in dehydration rate and QMs was encouraging to all staff. The Census varied widely from week to week due to COVID lockdowns and limits on admissions until the outbreak was resolved. While community nursing homes opened soon after vaccines became available, the VA maintained COVID restrictions the entire time of our study.

**Fig. 1:**
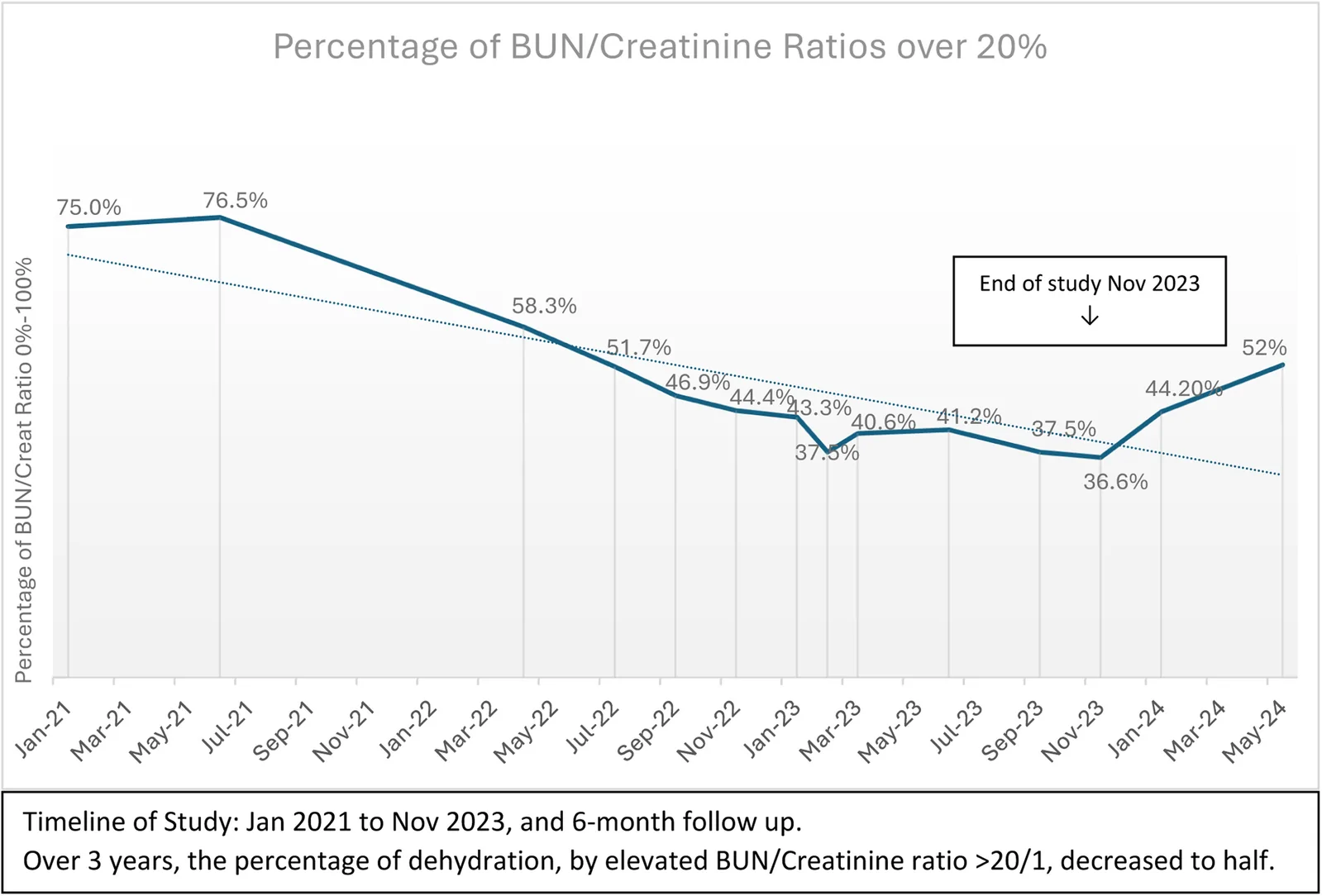
Timeline.

The prevalence of dehydration, defined as BUN/Cr ratio ≥20:1, decreased from 75.0% at baseline (January 2021) to 36.6% at study completion (November 2023), representing a 51% relative reduction. Figure 1 displays the complete timeline with 12 measurement points, showing relatively steady decline with some fluctuation during mid-2022 and early 2023. The most rapid improvement occurred during the first year of the intervention (75.0% to 51.7% by July 2022), with more gradual continued decline through 2023. During this same period, we observed marked improvements in multiple quality metrics previously associated with dehydration in the geriatric literature (Table). Fall rates declined from 6.4 in 2021 to 3.9 in 2023. Long-stay urinary tract infections decreased from 15 (FY2022) to 7 (FY2023). Catheter-associated UTI rates improved from 5th quintile to 1st quintile among VA nursing homes, achieving a rate of 0 per 1000 catheter-days. Facility-acquired MRSA infections similarly improved from 5th to 1st quintile. Hospitalizations per 1000 long-stay resident-days moved from 5th to 3rd quintile. For short-stay/rehabilitation residents, SAIL measures of functional improvement, rehospitalization rates, and successful discharge to community all showed consistent improvement over 11 quarters. Dehydration prevalence increased to 52% 6 months after completion of the study and remained at 53% two years after completion.

Dehydration is challenging to quantify. In older adults, physical findings are not reliable. We chose BUN/Creat ratio because of ease of access due to required periodic medication monitoring. Elevated sodium is also easily accessible and reliable for dehydration but can be complicated by medications that cause hyponatremia, especially in older residents. Residents with chronic renal failure (CRF) may have elevated creatinine, confounding the BUN/Creat ratio. Yet, we found that with hydration, first BUN decreased, followed by decrease in creatinine. This may have narrowed the ratio temporarily. Some residents’ elevated creatinine resolved, negating the diagnosis of CRF.

Challenges: Staff turnover requires us to continuously educate new staff. Resident turnover, co-morbidities, poor insight and unwillingness to drink require constant reminders from staff. Small resident numbers may not be representative or applicable to other facilities.

Residents with CRF may have persistently elevated creatinine, confounding the BUN/Cr ratio. We tracked all obtained BUN/Cr ratios in aggregate and did not exclude residents with known CRF, which may have inflated our baseline prevalence estimate and limited the specificity of BUN/Cr ratio for detecting true dehydration in this population. However, we observed that with hydration interventions, BUN decreased first, followed by decreases in creatinine, suggesting that at least some of the improvement reflected genuine hydration status changes rather than stable renal dysfunction. Notably, some residents' elevated creatinine resolved entirely during the study period, indicating that their baseline azotemia was prerenal and potentially reversible with hydration. Future studies should validate BUN/Cr ratio against gold-standard measures (serum osmolality) and stratify analyses by baseline kidney function.

Limitations: We are reporting the path and results for our VA facility, predominantly male, with small and fluctuating census. Pandemic-era timeframe (2021–2023), with changes in staffing, infection control, and transfer thresholds. We tracked all obtained BUN/Cr ratios of our residents in aggregate at the spot check times and did not exclude anyone or track individual residents. Although causality cannot be established in this single-site quality improvement initiative, increased attention to hydration may have contributed to observed improvements.

Large fluctuations of resident census due to lockdowns and reopenings and exceptionally long working hours with COVID, as well as staffing shortage due to illness, prevented us from tracking detailed resident data and reporting more granular baseline characteristics.

Future studies should evaluate whether increased education and focus on dehydration can improve the prevalence of dehydration and QMs in other facilities. Larger more detailed studies could identify the trajectory of individual responses to various approaches, as well as the reduction of the prevalence of chronic renal disease diagnoses with hydration.

Additionally, our use of BUN/Cr ratio ≥20:1 as the dehydration criterion, while pragmatic and clinically familiar, has not been validated against gold-standard measures (serum osmolality) in nursing home populations with high CRF prevalence. This limitation, combined with our inability to exclude residents with chronic renal disease from the aggregate data analysis, means our baseline prevalence estimate may not reflect the true burden of dehydration and our improvement may partially reflect changes in renal function rather than hydration status alone. Most importantly, the improvements observed in falls, UTIs, hospitalizations, and other quality metrics cannot be attributed causally to dehydration reduction in this uncontrolled, single-site study. These concurrent improvements may reflect secular trends, pandemic-related changes in infection control practices, Hawthorne effects from increased staff attention, or other facility-level quality improvement activities. Controlled trials with randomization, blinded outcome assessment, and measurement of mechanistic mediators (orthostatic vital signs, urine concentration, cognitive function) are required to establish whether dehydration reduction causally improves clinical outcomes.

### 5.1 Implications for Future Research

This quality improvement initiative establishes preliminary evidence that nursing home dehydration may be highly prevalent and modifiable through pragmatic interventions. However, several critical steps are required before definitive practice recommendations can be made. First, the BUN/Cr ratio should be validated against gold-standard hydration measures (serum osmolality) in nursing home populations, with stratification by chronic kidney disease status. Second, mechanistic studies should examine temporal relationships and dose-response effects, measuring whether dehydration improvement precedes and predicts reductions in falls, UTIs, and hospitalizations, and testing proposed causal pathways (e.g., hydration → improved orthostatic blood pressure regulation → reduced falls). Third, controlled trials are needed to establish causality; an initial pilot cluster-randomized trial (6-10 facilities) could test the standardized intervention bundle against enhanced usual care while measuring both hydration biomarkers and clinical outcomes. Such trials should include economic evaluation (cost per fall prevented, per hospitalization averted) to inform resource allocation decisions. If efficacy is demonstrated, a large, pragmatic trial across diverse nursing home settings would be warranted to establish generalizability and implementation strategies.

## 6 Conclusion

Both physiologic changes of normal aging and medications increase older adults’ risk for dehydration. Using education and a multi-pronged approach we reduced the prevalence of dehydration from 75% to 36.6%, and was associated with improvement of many QMs:

## Authorship

Dr. Hamrick conceived of the idea, designed the quality improvement project, and wrote the manuscript. M. Lisa Bauer gathered data and analyzed it. Both authors were involved in the roll-out of the project and in review of the manuscript.

## Ethical statement

Because we used anonymized data, we did not get consent and received IRB approval for non-human protocol #2023–0630.

## Data statement

The data is owned by the VA and we do not have the authority to share data.

## Declaration of generative AI use

AI was not used in the development of this manuscript.

## Fundings

This research did not receive any specific grant from funding agencies in the public, commercial, or not-for-profit sectors.

This material is the result of work supported with resources and the use of facilities at the Cincinnati VA Medical Center.

## CRediT authorship contribution statement

**Irene Hamrick:** Writing – review & editing, Writing – original draft, Supervision, Project administration, Methodology, Investigation, Formal analysis, Data curation, Conceptualization. **M. Lisa Bauer:** Writing – review & editing, Formal analysis, Data curation.

## Declaration of competing interest

The authors declare that they have no known competing financial interests or personal relationships that could have appeared to influence the work reported in this paper.
